# Synthesis and Evaluation of the Cytotoxic Activity of Water-Soluble Cationic Organometallic Complexes of the Type [Pt(η^1^-C_2_H_4_OMe)(L)(Phen)]^+^ (L = NH_3_, DMSO; Phen = 1,10-Phenanthroline)

**DOI:** 10.3390/pharmaceutics13050642

**Published:** 2021-04-30

**Authors:** Federica De Castro, Erika Stefàno, Danilo Migoni, Giorgia N. Iaconisi, Antonella Muscella, Santo Marsigliante, Michele Benedetti, Francesco P. Fanizzi

**Affiliations:** Dipartimento di Scienze e Tecnologie Biologiche ed Ambientali, Università del Salento, Prov.le Lecce-Monteroni, Centro Ecotekne, 73100 Lecce, Italy; federica.decastro@unisalento.it (F.D.C.); erika.stefano@unisalento.it (E.S.); danilo.migoni@unisalento.it (D.M.); giorgianatalia.iaconisi@studenti.unisalento.it (G.N.I.); antonella.muscella@unisalento.it (A.M.); santo.marsigliante@unisalento.it (S.M.); fp.fanizzi@unisalento.it (F.P.F.)

**Keywords:** cisplatin, coordination compounds, platinum complex, cationic complex, square planar complex, organometallic complex, antitumor drug, cytotoxicity, antitumor activity

## Abstract

Starting from the [PtCl(η^1^-C_2_H_4_OMe)(phen)] (phen = 1,10-phenanthroline, **1**) platinum(II) precursor, we synthesized and characterized by multinuclear NMR new [Pt(η^1^-C_2_H_4_OMe)(L)(phen)]^+^ (L = NH_3_, **2**; DMSO, **3**) complexes. These organometallic species, potentially able to interact with cell membrane organic cation transporters (OCT), violating some of the classical rules for antitumor activity of cisplatin analogues, were evaluated for their cytotoxicity. Interestingly, despite both complexes **2** and **3** resulting in greater cell uptake than cisplatin in selected tumor cell lines, only **3** showed comparable or higher antitumor activity. General low cytotoxicity of complex **2** in the tested cell lines (SH-SY5Y, SK-OV-3, Hep-G2, Caco-2, HeLa, MCF-7, MG-63, ZL-65) appeared to depend on its stability towards solvolysis in neutral water, as assessed by NMR monitoring. Differently, the [Pt(η^1^-C_2_H_4_OMe)(DMSO)(phen)]^+^ (**3**) complex was easily hydrolyzed in neutral water, resulting in a comparable or higher cytotoxicity in cancer cells with respect to cisplatin. Further, both IC_50_ values and the uptake profiles of the active complex appeared quite different in the used cell lines, suggesting the occurrence of diversified biological effects. Nevertheless, further studies on the metabolism of complex **3** should be performed before planning its possible use in tissue- and tumor-specific drug design.

## 1. Introduction

Cisplatin was synthesized for the first time in 1845, together with its *trans* isomer, by the Italian chemist Michele Peyrone. Its antitumor properties were highlighted only after 1960, thanks to the work of Barnett Rosenberg et al. [1,2]. Interestingly, a long time was required for its approval as an anticancer drug (in 1979) due to its relevant side effects, notwithstanding the generally observed increase of life expectancy or the complete recovery of oncological patients [3,4]. Another critical aspect of cisplatin was identified in its intrinsic low solubility, strongly limiting the possible pharmacological application with resistant tumors, probably requiring higher dosages. In any case, due to the progresses made in cisplatin therapeutic protocols, to date it remains one of the most widely used anticancer drugs [4,5]. For this reason, research on new platinum-based antitumor drugs has been mainly focused on the development of new complexes violating the classical rules early defined for antitumor activity of platinum compounds (i.e., *cis*-isomers, presence of two medium labile ligands and two inert *N*-donors different from tertiary amines) [4,5,6,7,8,9,10]. About cisplatin mechanism of action, it is well known that passive diffusion is not the only mechanism of cellular uptake, as several experiments showed that cisplatin is also imported by cell membrane carriers normally involved in the transport of different substrates. In this context, the two *cis* chlorido ligands of cisplatin, characterized by a medium lability, seem necessary to achieve the equilibrium formation of the antitumor active cationic solvento species *cis*-[PtCl(NH_3_)_2_(OH_2_)]^+^, *cis*-[Pt(NH_3_)_2_(OH_2_)_2_]^2+^, and *cis*-[Pt(NH_3_)_2_(OH)(OH_2_)]^+^. The peculiar ability of cisplatin to produce such cationic species once inside the cytoplasm, due to the relatively low chloride concentration with respect to blood plasma (extracellular plasma [Cl^−^] > 100 mM; cell cytoplasm [Cl^−^] ≈ 23 mM; cell nucleus ≈ 4 mM) [4,5,11] is one of the serendipity aspects that have determined the success of this drug. In fact, the formation of reactive charged platinum solvento species inside the blood plasma seems related to the severity of side effects observed elsewhere in the body [12,13]. Indeed, the unselective binding of such reactive hydrolysis products to serum proteins, etc., is considered responsible for the observed cisplatin toxicity and side effects. Moreover, the reactivity of a platinum drug in the blood serum is a key factor determining how much of the administered drug actually reaches the cell membranes to begin uptake via passive diffusion or active transport (involving hCtr1, OCT, etc.) [14,15]. Furthermore, the hydrolyzed drug, when formed inside the cell cytoplasm, constitutes the essential active species for the desired antitumor activity. These reactive forms seem capable to interact with suitable nucleophilic targets present in cells, such as nucleic acids, proteins, thiols, phospholipids, cytoskeleton, etc. Among them, DNA is considered the main pharmacological target, as its functionality results strongly altered by interaction with cisplatin [4,14,15,16]. Since its approval, to solve some of the problems occurred in clinical applications of cisplatin, many attempts have been made to develop new different antitumor active platinum-based drugs with a greater spectrum of efficacy, a different mechanism of action, lower side effects, and enhanced water solubility [4,5,17,18,19]. In particular, recent studies on the action mechanism of cisplatin and oxaliplatin showed that cell membrane organic cation transporters (OCT) can be involved in the uptake of platinum drugs in the form of cationic solvento species [20,21,22,23,24,25]. This was also confirmed by recent studies on new cationic Pt(II)-complexes showing a relevant antitumor activity [5,19,26,27].

It should be also underlined that there is a limited number of studies on platinum organometallic compounds tested as antitumor drugs [28,29,30,31,32,33,34,35]. In this regard, the Zeise’s anion, [PtCl_3_(η^2^-C_2_H_4_)]^−^, showing an extreme lability of the *trans* to olefin chlorido ligand and a peculiar reactivity of the coordinated ethene, resulted to be a suitable precursor for the synthesis of new antitumor organometallic complexes [19]. In fact, it allows the formation of platinum(II) organometallic complexes by the promotion of coordination in the platinum(II) sphere of specific carrier ligands and the exploitation of the intrinsic π-acid character of platinum-coordinated η^2^-ethene [36,37,38,39]. We already explored the synthetic possibilities offered by the peculiar reactivity of the Zeise’s anion in strongly basic oxydrilated solvents, demonstrating that in these conditions, anionic species of the type *trans*-[PtCl_2_(η^2^-C_2_H_4_)(OR)]^−^ (R = H, alkyl) are easily formed. Interestingly, the last product shows a quite different reactivity with respect to that of the Zeise’s anion, allowing the synthesis of [PtCl(η^1^-C_2_H_4_OR)(*N-N*)] (*N-N* = dinitrogen ligand) complexes. These include, besides the here considered [PtCl(η^1^-C_2_H_4_OMe)(phen)] (phen = 1,10-phenanthroline, **1**), also other complexes bearing even more hindered dinitrogen ligands [39].

The aim of this work was to synthesize and characterize new cationic Pt(II) complexes able to cross the cell membrane and exhibiting antitumor activity with mechanisms potentially different from that of cisplatin. In this way, we planned to change and possibly improve the selectivity and efficacy toward tumor cells, with eventual reduction of side effects. In particular, we focused on Pt(II)-phen derivatives, which can also be synthesized in high yields, Ref. [39] targeting new water-soluble cationic coordination compounds of the type [Pt(η^1^-C_2_H_4_OR)(L)(phen)]^+^ (L = medium labile or not labile substituent; R = alkyl). The specific interest toward this type of complexes relies on their potential ability to interact with organic cation transporters [20,21,22,23,24,25] and to intercalate DNA and other nucleic acids, as generally observed in the case of phen derivatives [40,41]. These complexes were obtained and tested as new potential Pt-based antitumor drugs, as described in the present work.

## 2. Materials and Methods

### 2.1. Reagents and Methods

Commercially available reagents and solvents were used as received, without further purification. Zeise’s salt, K[PtCl_3_(η^2^-C_2_H_4_)]·H_2_O, was synthesized with a previously reported procedure, as indicated in ref. [39]. Elemental analyses were performed with a CHN Eurovector EA 3011. NMR spectra were recorded with Bruker Avance III 400 and 600 NMR spectrometers, equipped with probes for inverse detection and with *z* gradient for gradient-accelerated NMR spectroscopy. ^1^H NMR monodimensional spectra and [^1^H,^195^Pt]-HETCOR bidimensional experiments were recorded by using CDCl**_3_** or D_2_O as solvents. ^1^H NMR spectra were referenced to TMS; the residual proton signal of the solvent [CDCl**_3_**; δ(^1^H) = 7.24 ppm**;** D_2_O; δ(^1^H) = 4.7 ppm] was used as the internal standard. ^195^Pt NMR chemical shifts were referenced to H_2_[PtCl_6_] [δ(^195^Pt) = 0 ppm] in D_2_O, as the external reference.

### 2.2. Synthesis of Pt(II) Complexes

[PtCl(η^1^-C_2_H_4_OMe)(phen)] (**1**). Na (81 mg, 3.5 mmol) was dissolved in 3.5 mL of MeOH (−20 °C, ice-NaCl bath). After evolution of H_2_, the temperature was increased to 0 °C (ice bath), then the Zeise’s salt, K[PtCl_3_(η^2^-C_2_H_4_)]·H_2_O, (100 mg, 0.26 mmol) was added under magnetic stirring. A white precipitate of KCl was immediately formed, and the color of the solution changed from bright to pale yellow. After about 2 min from dissolution, 1,10-phenanthroline (47 mg, 0.26 mmol) was added to the reaction mixture, maintaining a vigorous stirring. After a few more minutes, the formation of a yellow precipitate, poorly soluble in cold methanol, started. The reaction was then followed by ^1^H NMR spectroscopy, by analyzing 10 μL aliquots of the rection suspension after dissolution in an NMR tube containing 500 μL of CD_3_OD. In this way, the ^1^H NMR signals of the final product **1**, the free phen ligand, and the *trans*-[PtCl_2_(η^2^-C_2_H_4_)(OMe)]^−^ intermediate species, could be observed. The three specie’s relative amount changed with time in favor of complex **1**, which was the only remaining precipitate at the end of the reaction, after about 4 h. The final product was first isolated by filtration, then washed with abundant water (to eliminate excess base and formed NaCl), and finally dried under vacuum. Complex **1** yield 115 mg, 95% referred to platinum. Anal. Calcd. for C_15_H_15_ClN_2_OPt: C, 38.3; H, 3.2; N, 6.0%. Found: C, 38.0; H, 3.1; N, 6.1%. NMR (400 MHz, CDCl_3_, 300 K). δ(^1^H) 2.41 (m, 2H, Pt-C_α_H_2_, ^2^*J*_Pt–H_ = 90 Hz), 3.42 (s, 3H, OCH_3_), 3.70 (m, 2H, C_β_H_2_-O), 7.81 (d.d., 1H, CH), 7.95 (m, 3H, CH), 8.55 (d, 1H, CH), 8.65 (d, 1H, CH), 9.53 (d, 1H, CH, ^3^*J*_Pt-H_ = 57 Hz) and 9.81 (d, 1H, CH) ppm; δ(^195^Pt) −3243 ppm.

[Pt(η^1^-C_2_H_4_OMe)(NH_3_)(phen)]Cl (**2**). In a typical reaction, the solid complex **1** (50 mg, 0.11 mmol) was suspended under stirring in 2.5 mL of 30% NH_3_ in water (m/m), at room temperature. After about two days, a colorless solution was obtained, which provided the final [Pt(η^1^-C_2_H_4_OMe)(NH_3_)(phen)]Cl (**2**) complex salt, after evaporation under vacuum to eliminate water and excess NH_3_. Complex **2** yield 52 mg, 99.9%. Anal. Calcd. for C_15_H_18_ClN_3_OPt: C, 37.0; H, 3.7; N, 8.6%. Found: C, 36.9; H, 3.9; N, 8.5%. NMR (400 MHz, D_2_O/H_2_O = 10/90, 300 K): δ(^1^H) 1.46 (m, 2H, Pt-C_α_H_2_, ^2^*J*_Pt–H_ = 50 Hz), 3.38 (s, 3H, OCH_3_), 3.37 (m, 2H, C_β_H_2_-O), 4.20 (s, 3H, Pt-NH_3_), 7.31 (d, 1H, CH), 7.37 (d, 1H, CH), 7.47 (d.d., 1H, CH), 7.66 (d.d., 1H, CH), 8.19 (d, 1H, CH), 8.27 (d, 1H, CH), 8.32 (d, 1H, CH), 8.38 (d, 1H, CH); δ(^195^Pt) −3322 ppm.

[Pt(η^1^-C_2_H_4_OMe)(DMSO)(phen)]Cl (**3**). In a typical reaction, the solid complex **1** (50 mg, 0.105 mmol) was suspended in a DMSO (10 mL) under stirring. The reaction was nearly quantitative, and after 2 days at room temperature, a yellow DMSO solution containing the final pure [Pt(η^1^-C_2_H_4_OMe)(DMSO)(phen)]Cl (**3**) complex salt was obtained. Complex **3**·2DMSO could be isolated as a microcrystalline precipitate by adding a mixture of ethanol/diethylether = 1:1 to a concentrated DMSO solution, followed by filtration. The obtained complex was then washed with the same non-solvent mixture and dried. Isolated yield 42 mg, 57%. Anal. Calcd. for C_21_H_33_ClN_2_O_4_PtS_3_: C, 35.8; H, 4.7; N, 4.0%. Found: C, 35.9; H, 4.8; N, 3.9%. NMR (400 MHz, D_2_O, 300 K): δ(^1^H) 1.74 (m, 2H, Pt-C_α_H_2_, ^2^*J*_Pt–H_ = 78 Hz), 3.37 (s, 3H, OCH_3_), 3.59 (m, 2H, C_β_H_2_-O), 3.68 (s, 6H, Pt-DMSO, ^3^*J*_Pt–H_ = 31 Hz), 8.00 (d, 1H, CH), 8.06 (s, 1H, CH), 8.07 (s, 1H, CH), 8.09 (d, 1H, CH), 8.75 (d.d., 1H, CH), 8.83 (d.d., 1H, CH), 9.03 (d, 1H, CH), 9.70 (d, 1H, CH); δ(^195^Pt) −3988 ppm.

### 2.3. Cell Cultures

The human cancer cell lines used for the evaluation of the Pt compounds cytotoxicity (see Table 1) were cultured in different culture media, as reported below:

- Caco-2: DMEM (low glucose) medium (Sigma-Aldrich, St. Louis, MO, USA), 10% FBS (Sigma-Aldrich, St. Louis, MO, USA), glutamine 2 mM, penicillin (100 U/mL), streptomycin (100 mg/mL), 1% non-essential amino acids.
- MG-63 and SK-OV-3: DMEM (high glucose) medium (EuroClone, Pero, MI, Italy), 10% FBS (Sigma-Aldrich, St. Louis, MO, USA), glutamine 2 mM, penicillin (100 U/mL), streptomycin (100 mg/mL).
- HeLa, MCF-7 and ZL-55: RPMI 1640 (EuroClone, Pero, MI, Italy), 10% FBS (Sigma-Aldrich, St. Louis, MO, USA), glutamine 2 mM, penicillin (100 U/mL), streptomycin (100 mg/mL).
- Hep-G2: DMEM (low glucose) medium (Sigma-Aldrich, St. Louis, MO, USA), 10% FBS (Sigma-Aldrich, St. Louis, MO, USA), glutamine 2 mM, penicillin (100 U/mL), streptomycin (100 mg/mL).
- SH-SY5Y: 1:1 mixture of DMEM (high glucose) and Ham’s F-12 Nutrient Mixture (Sigma-Aldrich, St. Louis, MO, USA), 10% FBS (Sigma-Aldrich, St. Louis, MO, USA), glutamine 2 mM, penicillin (100 U/mL), streptomycin (100 mg/mL).

### 2.4. Cytotoxicity Assays

We evaluated the IC_50_ with 3-(4,5-dimethylthiazol-2-yl)-2,5-diphenoltetrazolium bromide (MTT) and sulforhodamine B (SRB) assays. The SRB assay and the conversion of MTT by cells were both used as an indicator of cell number, as described previously [42]. Cells, at 70–80% confluency, were trypsinized (0.25% trypsin with 1 mM EDTA), washed, and resuspended in growth medium. Then, 100 μL of a cell suspension (8 × 10^4^ cells/mL) was added to each well of a 96-well plate. After overnight incubation, cells were treated with different Pt compounds for 12–72 h. The percentage cell survival was calculated as the absorbance ratio between treated and untreated cells (medium-treated control cell). Viable cells were also counted by the Trypan blue exclusion assay and light microscopy. The data presented are means standard deviation (S.D.) from eight replicate wells per microliter plate, repeated three times.

### 2.5. Analysis by ICP-AES (Inductively Coupled Plasma Atomic Emission Spectroscopy)

For the determination of platinum concentration, each sample (consisting of cellular pellet) was previously treated with 0.5 mL of 67% super-pure nitric acid at room temperature for 24 h, to obtain a clear solution. Acid digestion was required to destroy the complex organic matrix and obtain the complete mineralization of the samples, essential for subsequent determinations. The samples were then diluted to a final volume of 5 mL, in order to obtain a suitable concentration of the acid used in the mineralization process and avoid damage to the system. Each sample was filtered before analysis (0.45 μm) to prevent the entry of any remaining suspension in the measuring instrument. The platinum concentration in the analyzed samples was determined by a Thermo iCAP 6000 spectrometer. The spectrophotometer was calibrated with a calibration line consisting of four points, each corresponding to a concentration of the element: 1 μg/L, 10 μg/L, 100 μg/L, 1000 μg/L.

## 3. Results and Discussion

### 3.1. PtCl(η^1^-C_2_H_4_OMe)(phen)] *(**1**)*, phen = 1,10-phenanthroline

**Scheme 1:**
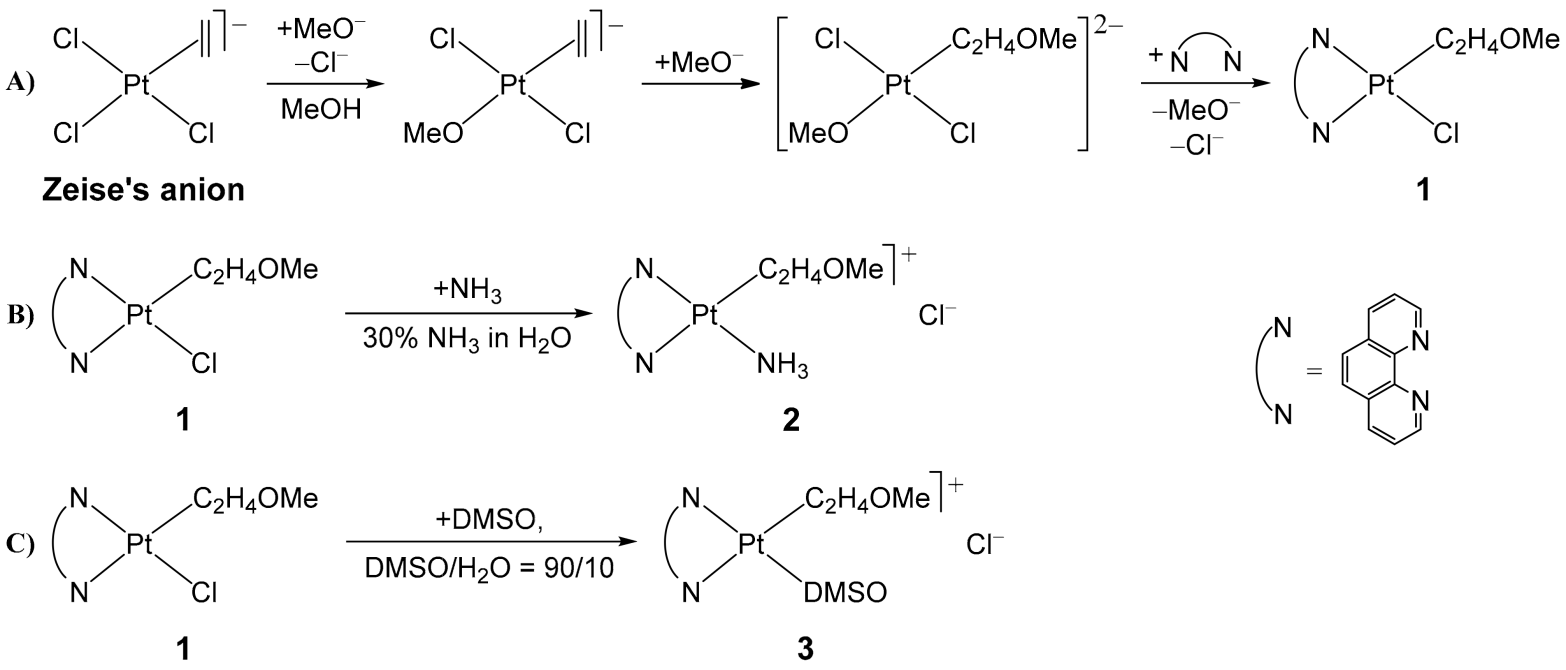
(**A**) Synthesis of the [PtCl(η^1^-C_2_H_4_OMe)(phen)] (**1**) complex precursor, starting from the Zeise’s anion, dissolved in strongly basic MeOH, and the phen ligand. (**B**) Synthesis of the [Pt(η^1^-C_2_H_4_OMe)(NH_3_)(phen)]^+^ (**2**) complex starting from **1** and NH_3_. (**C**) Synthesis of the [Pt(η^1^-C_2_H_4_OMe)(DMSO)(phen)]^+^ (**3**) complex starting from **1** and DMSO.

We obtained the solid [PtCl(η^1^-C_2_H_4_OMe)(phen)] (**1**) complex as a precipitate, by following a previously reported synthetic procedure [39,43]. The Zeise’s salt, K[PtCl_3_(η^2^-C_2_H_4_)]·H_2_O, was dissolved in strongly basic methanol ([NaOMe] ≈ 1M) before addition of the stoichiometric amount of phen. In this way, the neutral [PtCl_2_(η^2^-C_2_H_4_)(phen)] pentacoordinate complex, normally produced when the phen ligand is added to a methanol solution of the Zeise’s salt [44], was not observed. The absence of pentacoordinate species in the presence of excess NaOMe was due to both its high reactivity in the adopted experimental conditions and the preliminary formation of the *trans*-[PtCl_2_(η^2^-C_2_H_4_)(OMe)]^−^ anionic complex which is characterized by a lower reactivity with respect to *N*-donors [39]. This reaction mechanism (Scheme 1) was also confirmed in the present case by ^1^H NMR monitoring of complex **1** formation reaction.

### 3.2. Synthesis of [Pt(η^1^-C_2_H_4_OMe)(NH_3_)(phen)]Cl *(**2**)* and [Pt(η^1^-C_2_H_4_OMe)(DMSO)(phen)]Cl *(**3**)* Complexes

In this work, we evaluated the possibility to synthesize water-soluble phenanthroline cationic complexes suitable for exhibiting antitumor activity, as previously observed in the case of similar [PtCl(η^2^-C_2_H_4_)(*N-N*)]^+^, *N-N* = (*R*,*R*) and (*S*,*S*)-1,2-diaminocyclohexane, cationic species [19]. These latter compounds were also suggested as possible prodrugs introducing the active Pt(1*R*,2*R*-diaminocyclohexane) moiety in the cells. Unfortunately, due to the extremely high tendency to lose the η^2^-ethene ligand in the presence of nucleophilic agents (as the simple Cl^−^ ligand), the analogous [PtCl(η^2^-C_2_H_4_)(phen)]^+^ complex results unsuitable for a slow release of the Pt(phen) moiety in physiological conditions. On the other hand, also the direct use of [PtCl(η^1^-C_2_H_4_OMe)(phen)] (**1**), as precursor of the olefin cationic species [39,43], for antitumor activity evaluations, was prevented due to its extremely low water solubility. Therefore, we decided to synthesize and test new types of water-soluble cationic species of the type [Pt(η^1^-C_2_H_4_OMe)(L)(phen)]^+^ obtained starting from complex **1**, after chlorido substitution with a neutral L ligand. Such new species are expected to be water-soluble prodrugs, possible sources of Pt(L)(phen) and Pt(phen) active moieties, and suitable for antitumor activity evaluation. For this reason, in the present study we focused on the synthesis and biological activity evaluation of two [Pt(η^1^-C_2_H_4_OMe)(L)(phen)]Cl complexes as prototypes of those containing, besides the chelated phen and the η^1^-C_2_H_4_OMe, a further nitrogen (L = NH_3_) or sulfur (L = DMSO) ligand in the Pt(II) coordination sphere.

### 3.3. Pt(η^1^-C_2_H_4_OMe)(NH_3_)(phen)]Cl *(**2**)*

Complex **2** could be obtained by reacting the solid [PtCl(η^1^-C_2_H_4_OMe)(phen)] (**1**) complex suspended in a concentrated water solution of ammonia. Quantitative substitution of the chlorido ligand with NH_3_ took place, leading to formation of the colorless water-soluble [Pt(η^1^-C_2_H_4_OMe)(NH_3_)(phen)]Cl (**2**) complex that could be isolated and characterized by NMR spectroscopy.

**Figure 1:**
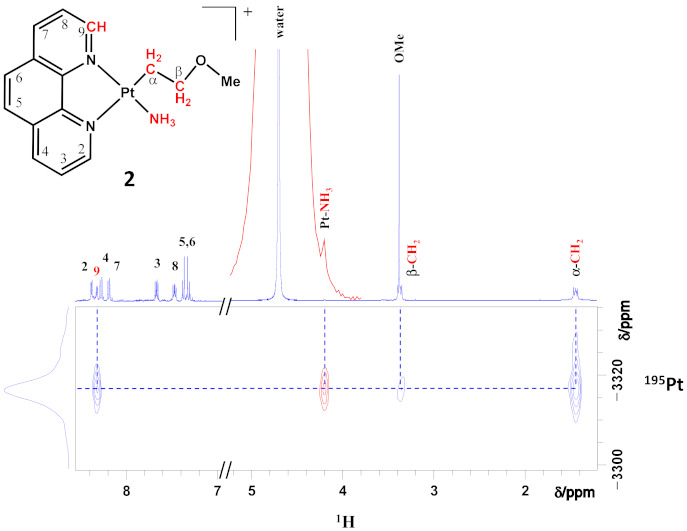
[^1^H,^195^Pt]-HETCOR NMR spectrum of the [Pt(η^1^-C_2_H_4_OMe)(NH_3_)(phen)]Cl (**2**) complex dissolved in D_2_O. In the above-reported schematic molecular structure, the groups whose H atoms ^1^H NMR signals are coupled with the ^195^Pt are evidenced in red. When a H_2_O/D_2_O = 90:10 solvent mixture was used, a new cross peak, corresponding to the ^1^H NMR signal of the Pt-bonded NH_3_ group (indicated in red together with the corresponding ^1^H NMR signal produced in the 1D ^1^H NMR spectrum) appeared.

The observed δ(^195^Pt) NMR signal exhibited by complex **2** corresponds to a chemical shift about 80 ppm lower, with respect to that of complex **1**, as expected on the basis of the slightly higher NMR shielding ability of a coordinated ammonia with respect to a chlorido ligand [45]. The bidimensional [^1^H,^195^Pt]-HETCOR spectrum, highlighting the cross peaks, accounts for the NMR couplings between phenanthroline H9 (close to the N10 which is in *trans* to NH_3_), coordinated NH_3_, σ-bonded C_α_H_2_, C_β_H_2_, and the central ^195^Pt (evidenced in red, in the schematic representation of the molecular structure reported in Figure 1). No cross peak was observed for the phen H(2) proton (close to the N1 *cis* to NH_3_), as expected for the lengthening of the Pt–N bond *trans* to the σ-alkyl. Complex **2** resulted to be very stable in water solution for more than 15 days, as demonstrated by the absence of any ^1^H NMR signals variation observed in a neutral D_2_O/H_2_O = 10:90 solution.

### 3.4. Pt(η^1^-C_2_H_4_OMe)(DMSO)(phen)]Cl *(**3**)*

When the solid complex **1** was reacted with excess DMSO, the quantitative substitution of the coordinated chlorido ligand occurred, producing the [Pt(η^1^-C_2_H_4_OMe)(DMSO)(phen)]Cl (**3**) complex, as observed by ^1^H NMR monitoring. Complex **3** could be isolated at room temperature by ethanol/diethyl ether addition to a concentrated DMSO solution.

A ^195^Pt NMR chemical shift decrease of about 800 ppm was observed, going from complex **1** to **3**. Consistently, a similar ^195^Pt NMR chemical shift decrease was observed, going from the *cis*-[PtCl_2_{1,4-(*i*-Pr)_2_-dab}] complex, δ(^195^Pt) = −2300 ppm, to the *cis*-[PtCl{1,4-(*i*-Pr)_2_-dab}(DMSO)]Cl complex, δ(^195^Pt) = −3150 ppm, with substitution of the coordinated chlorido ligand with a DMSO [46].

**Figure 2:**
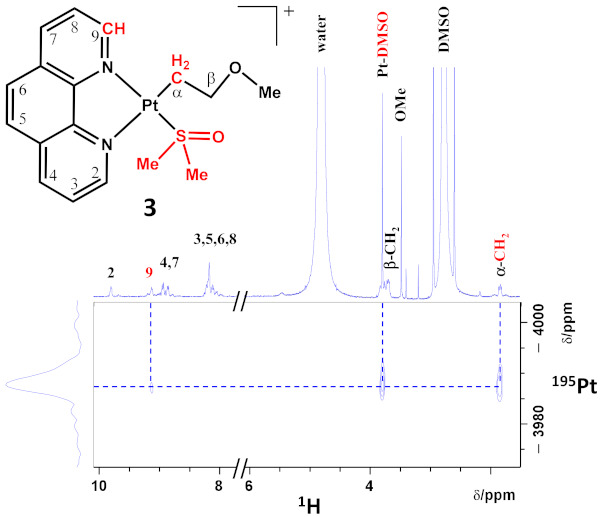
[^1^H,^195^Pt]-HETCOR NMR spectrum of the [Pt(η^1^-C_2_H_4_OMe)(DMSO)(phen)]Cl (**3**) complex dissolved in a D_2_O/DMSO = 98:2 mixture. In the above-reported schematic molecular structure, the groups whose H atoms ^1^H NMR signals are coupled with the ^195^Pt are evidenced in red.

The two-dimensional [^1^H,^195^Pt]-HETCOR spectrum of a freshly prepared solution of complex **3** (≈0.3 mM in D_2_O/DMSO = 98:2 mixture, pH ≈ 7) with highlighted ^1^H-^195^Pt cross peaks is shown in Figure 2. The observed cross peaks account for the NMR couplings between phenanthroline H9 (close to the N10 which is in *trans* to DMSO), coordinated DMSO, σ-bonded C_α_H_2_, and the central ^195^Pt (evidenced in red, in the schematic representation of the molecular structure reported in Figure 2). No cross peak was observed for the phen H(2) proton (close to the N1 *cis* to DMSO), as expected for the lengthening of the Pt-N bond *trans* to the σ-alkyl.

**Scheme 2:**
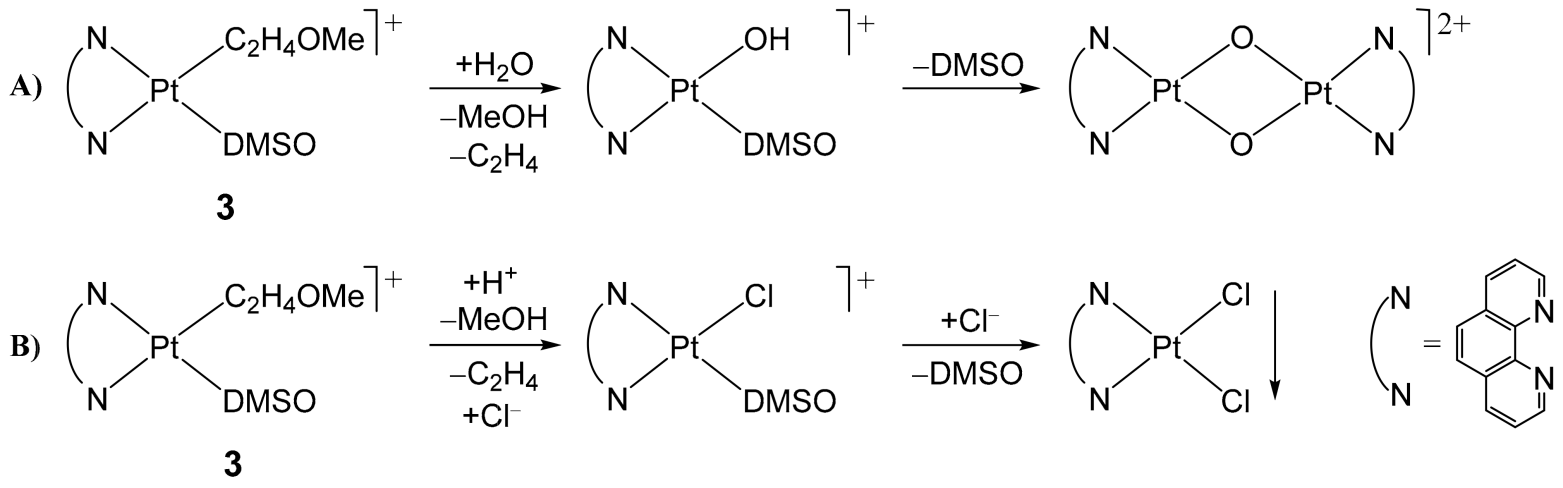
(**A**) Hydrolysis in water of the [Pt(η^1^-C_2_H_4_OMe)(DMSO)(phen)]^+^ (**3**) complex, involving the σ-C_2_H_4_OMe moiety, providing the reactive [Pt(DMSO)(OH)(phen)]^+^ intermediate, followed by slow DMSO loss and formation of the [{Pt(μ-OH)(phen)}_2_]^2+^ hydroxo-bridged dimer. The last step is disfavored by a high DMSO concentration. (**B**) Parallel mechanism of water hydrolysis for complex **3**, favored by high Cl^−^ concentration, providing the reactive [PtCl(DMSO)(phen)]^+^ intermediate, producing by DMSO loss, the low soluble [PtCl_2_(phen)] species.

As observed by ^1^H NMR monitoring in neutral D_2_O solution, in sharp contrast with respect to **2**, complex **3** slowly underwent ethene and methanol release and further DMSO dissociation when dissolved in water, even in the presence of DMSO (Figure S1). As a consequence, besides the hydrolysis of the σ-C_2_H_4_OMe moiety, with release of ethane and methanol, the formation of hydrolytic products was also observed. In particular, NMR spectral data and literature evidence of the high acidity of Pt coordinated water in these systems, suggests the formation of [Pt(DMSO)(OH)(phen)]^+^ and [{Pt(μ-OH)(phen)}_2_]^2+^ complexes with an asymmetric and a symmetric set of phenanthroline ^1^H NMR signals, respectively. Both the new [Pt(DMSO)(OH)(phen)]^+^ and the already known [{Pt(μ-OH)(phen)}_2_]^2+^ complexes [47] were spectroscopically identified (Figure S1), in the latter case also by ^195^Pt NMR spectroscopy (Figure S2). A mechanism for the observed hydrolysis process is reported in Scheme 2. During hydrolysis, the formation of slight amounts of the insoluble [PtCl_2_(phen)] complex, separating as a precipitate, was also observed. Therefore, for further biological studies, complex **3** was prepared in and used from DMSO/H_2_O = 90:10 stock solutions where it demonstrated a long-term stability (over 2 weeks at room temperature). It should be noted that, despite the observed decomposition of **3** in D_2_O, a small amount of water in the presence of excess DMSO disfavored complex **3** decomposition by chlorido reentering in the Pt coordination sphere and increased complex solubility in the stock solution. Complex **3** resulted indefinitely stable when stocked at −20 °C in DMSO/H_2_O = 90:10 mother solutions at a [3] ≈10 mM.

NMR monitoring demonstrated that complex **3** complete decomposition in neutral water was slow (about three days) and occurred via formation of solvento species of the type [Pt(DMSO)(OH)(phen)]^+^ and [{Pt(μ-OH)(phen)}_2_]^2+^, together with slight amounts of the insoluble [PtCl_2_(phen)] compound. These latter could therefore be considered as the possibly involved active species produced when diluting in biological assays DMSO/H_2_O = 90:10 stock solutions of complex **3**.

### 3.5. Cytotoxicity of the Potential Antitumor Drugs [Pt(η^1^-C_2_H_4_OMe)(NH_3_)(phen)]^+^ *(**2**)* and [Pt(η^1^-C_2_H_4_OMe)(DMSO)(phen)]^+^ *(**3**)*

Cells were treated with various concentrations (0.1–200 μM) of **2** or **3** in comparison with cisplatin, and viable cell number was determined by MTT metabolic assay and confirmed by SRB assay 12, 24, 48 and 72 h later.

For all cell lines, viable cell number values obtained after [Pt(η^1^-C_2_H_4_OMe)(DMSO)(phen)]^+^ treatment were corrected for a “blank” value (‘blank’ was the mean optical density of the background control wells containing the incubation medium and 0.725% DMSO, representing the highest DMSO quantity present in complex **3** solution). Furthermore, cell viability after 0.725% DMSO treatments of SK-OV-3, Hep-G2, and SH-SY5Y cell lines did not change significantly over 0–72 h incubation times and is shown in Figure S3.

**Figure 3:**
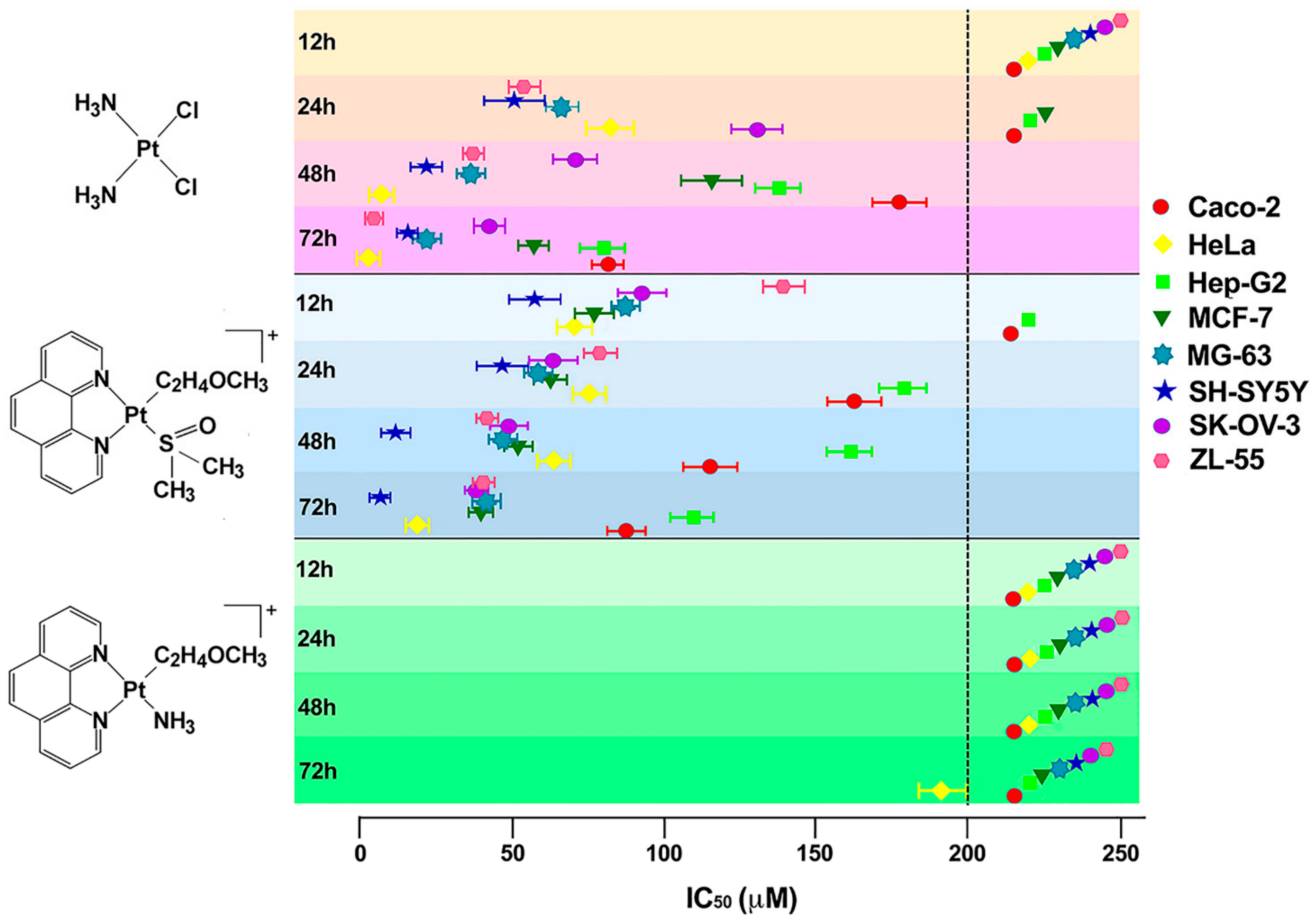
IC_50_ after 12, 24, and 48 h of treatment with [Pt(η^1^-C_2_H_4_OMe)(DMSO)(phen)]^+^, [Pt(η^1^-C_2_H_4_OMe)(NH_3_)(phen)]^+^ and cisplatin at different concentrations (0.1, 1, 10, 100, and 200 μM) obtained in different human cancer cell lines.

Of the two new organometallic Pt(II)-complexes containing 1,10-phenantroline, only complex **3** proved to be more effective than cisplatin in many of the cell lines used. In fact, it was possible to calculate the IC_50_ for compound **2** only in HeLa cells and after 72 h of incubation (190.9 ± 8.2 μM), as shown in Figure 3; in all the other cell lines and for all the incubation times tested, it was not possible to determine an IC_50_ value. It is generally known that the presence of DMSO causes a reduction of cytotoxicity or complete inactivation of most of the tested antitumor drugs [46,47,48,49]. Interestingly, we found that in the case of complex **3**, when administered to tumor cells, the presence of DMSO conferred a level of cytotoxicity comparable to that of cisplatin, rather than inactivating the molecule. This was probably due to the hydrolysis mechanism involving first the loss of the η^1^-C_2_H_4_OMe moiety and further the generation of active hydrolytic species exhibiting a cytotoxicity comparable to that of cisplatin.

**Figure 4:**
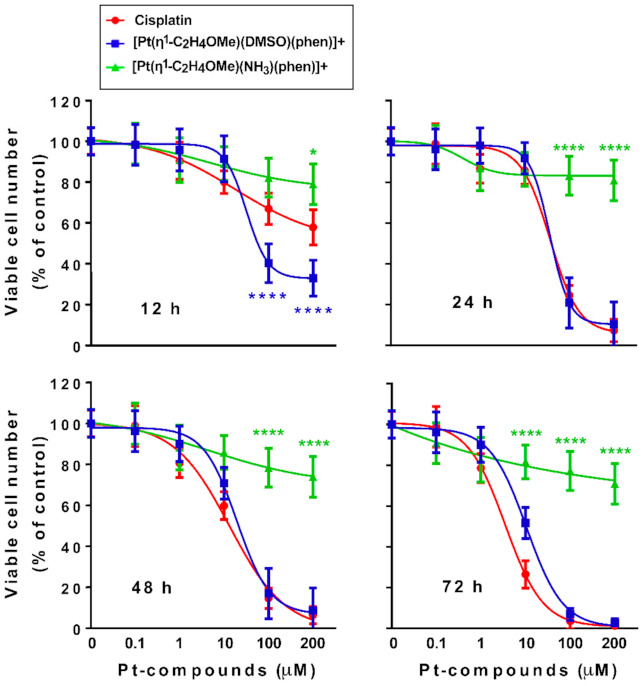
Cell viability assay after [Pt(η^1^-C_2_H_4_OMe)(DMSO)(phen)]^+^, [Pt(η^1^-C_2_H_4_OMe)(NH_3_)(phen)]^+^, and cisplatin treatments in SH-SY5Y cell line. The viability of the neuroblastoma cell line was evaluated after treatment with Pt compounds at different concentrations (0.1, 1, 10, 100, and 200 μM) for 12, 24, 48, and 72 h. The data are means ± S.D. of three different experiments run in eight replicates and are presented as percent of control. Asterisks indicate values that are significantly different (* *p* < 0.05; **** *p* < 0.0001) from those of cisplatin at the same concentrations and time points.

**Figure 5:**
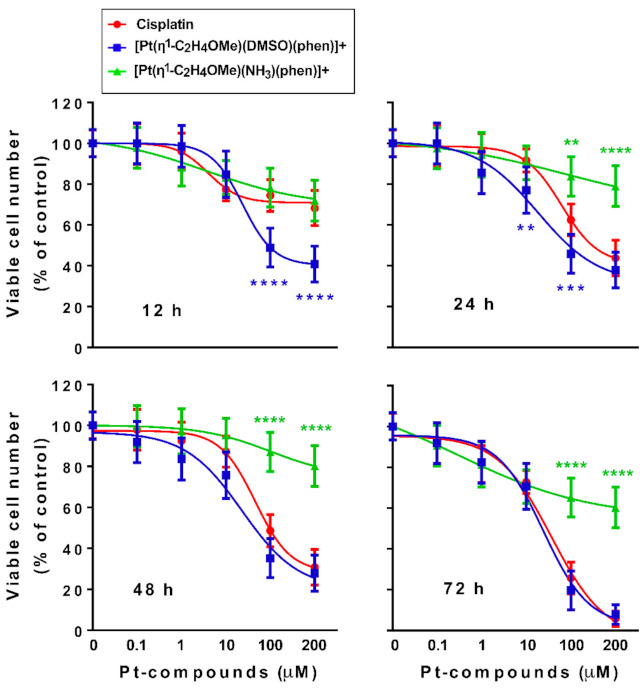
Cell viability assay after [Pt(η^1^-C_2_H_4_OMe)(DMSO)(phen)]^+^, [Pt(η^1^-C_2_H_4_OMe)(NH_3_)(phen)]^+^, and cisplatin treatments in SK-OV-3 cell line. The viability of the ovarian adenocarcinoma cell line was evaluated after treatment with Pt compounds at different concentrations (0.1, 1, 10, 100 and 200 μM) for 12, 24, 48, and 72 h. The data are means ± S.D. of three different experiments run in eight replicates and are presented as percent of control. Asterisks indicate values that are significantly different (** *p* < 0.01; *** *p* < 0.001; **** *p* < 0.0001) from those of cisplatin at the same concentrations and time points.

**Figure 6:**
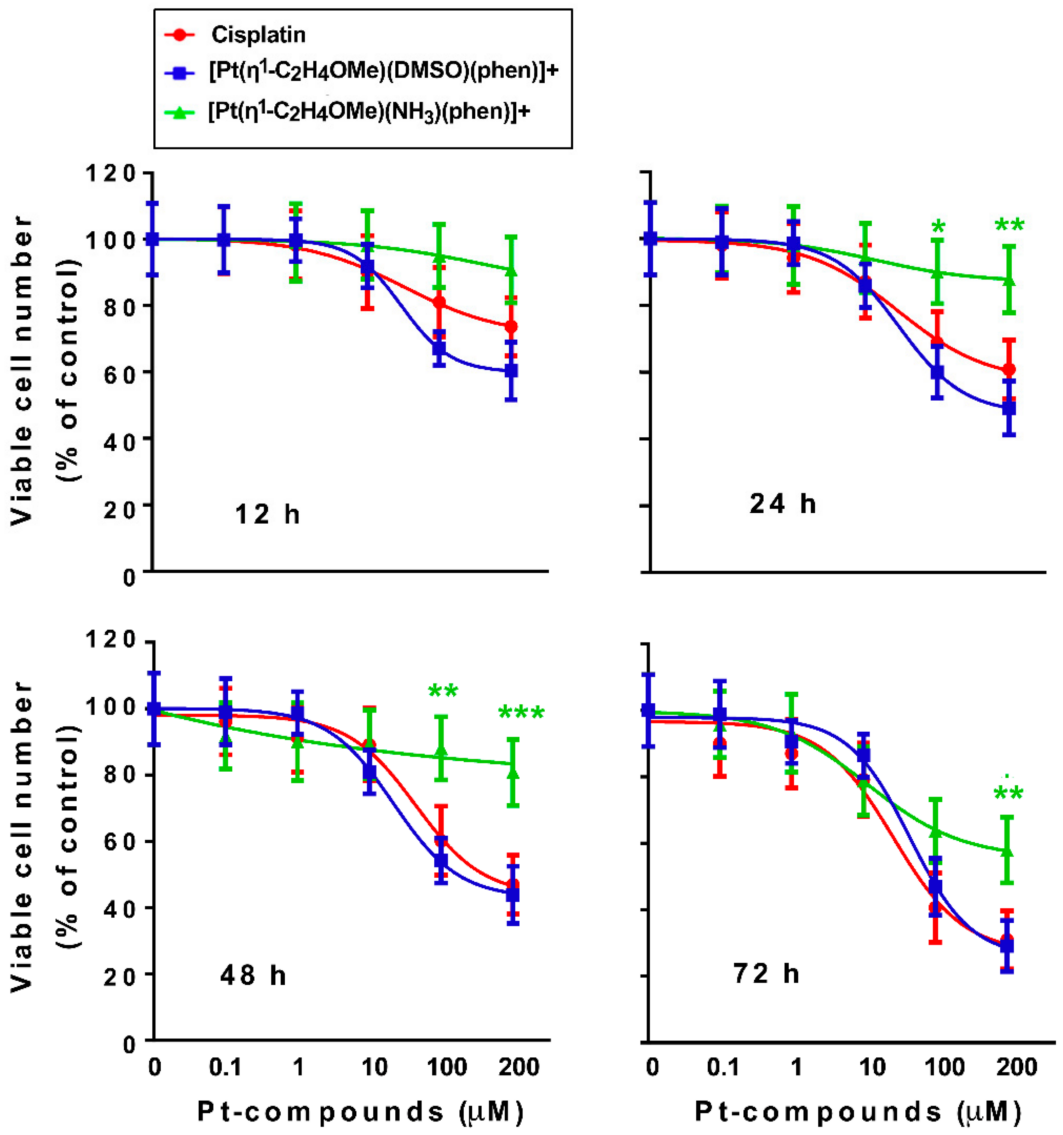
Cell viability assay after [Pt(η^1^-C_2_H_4_OMe)(DMSO)(phen)]^+^, [Pt(η^1^-C_2_H_4_OMe)(NH_3_)(phen)]^+^, and cisplatin treatments in Hep-G2 cell line. The viability of the hepatocellular cell line was evaluated after treatment with Pt compounds at different concentrations (0.1, 1, 10, 100, and 200 μM) for 12, 24, 48, and 72 h. The data are means ± S.D. of three different experiments run in eight replicates and are presented as percent of control. Asterisks indicate values that are significantly different (* *p* < 0.05; ** *p* < 0.01; *** *p* < 0.001) from those of cisplatin at the same concentrations and time points.

Conversely, [Pt(η^1^-C_2_H_4_OMe)(DMSO)(phen)]^+^ (**3**) showed higher cytotoxic effects than cisplatin in six of the eight cell lines treated, with an IC_50_ evaluable already at 12 h of incubation (Figure 3 and Table S1). IC_50_ at 12 h ranged between 56.9 ± 7.7 μM (SH-SY5Y cells) and 139.1 ± 6.8 μM (ZL-55 cells), and IC_50_ at 24 h ranged between 45.8 ± 7.8 μM (SH-SY5Y cells) and 178.5 ± 9.3 μM (Hep-G2 cells) (Table S1). Compared to cisplatin, a drastic reduction of cell viability after treatment with **3** was observed mainly in neuroblastoma (SH-SY5Y); no significant differences between cisplatin and complex **3** were observed in Hep-G2 cells. Furthermore, an intermediate sensitivity to **3** was shown by SK-OV-3 cells (Figure 3 and Table S1). The comparisons between the mortality curves of cisplatin, [Pt(η^1^-C_2_H_4_OMe)(NH_3_)(phen)]^+^ (**2**), and [Pt(η^1^-C_2_H_4_OMe)(DMSO)(phen)]^+^ (**3**) at various concentrations and at different incubation times for SH-SY5Y, SK-OV-3, and Hep-G2 cells are shown in Figure 4, Figure 5 and Figure 6, respectively.

As it can be seen, in SH-SY5Y cells, complex **3** was significantly more cytotoxic than cisplatin up to 24 h of treatment (IC_50_ between 56.9 ± 7.7 μM and 45.8 ± 7.8 μM), and at much longer times (72 h) the effects were the same (Figure 4). In SK-OV-3 cells, complex **3** caused a halving of the cell population rather early (12–24 h) (IC_50_ between 92.1 ± 7.8 μM and 62.8 ± 6.6 μM). By contrast, the IC_50_ was evaluable with cisplatin starting from 24 h of treatment (IC_50_ 130 ± 8.9 μM). However, also in this cell line, no significant differences were observed between **3** and cisplatin at very long incubation times (72 h) (IC_50_ 37.5 ± 3.2 μM and 41.7 ± 4.6 μM, respectively) (Figure 5).

With regard to Hep-G2 cells, there were no differences between the effects of **3** and those of cisplatin at any of the times tested and for any concentration in the 0.1–200 µM range (Figure 6).

Then, we established whether the different sensitivity of the three cell lines mentioned above (Hep-G2, SH-SY5Y, and SK-OV-3) depended on a different intracellular accumulation of the Pt(II) compounds.

### 3.6. In-Cell Accumulation of the Tested Pt(II) Compounds

**Figure 7:**
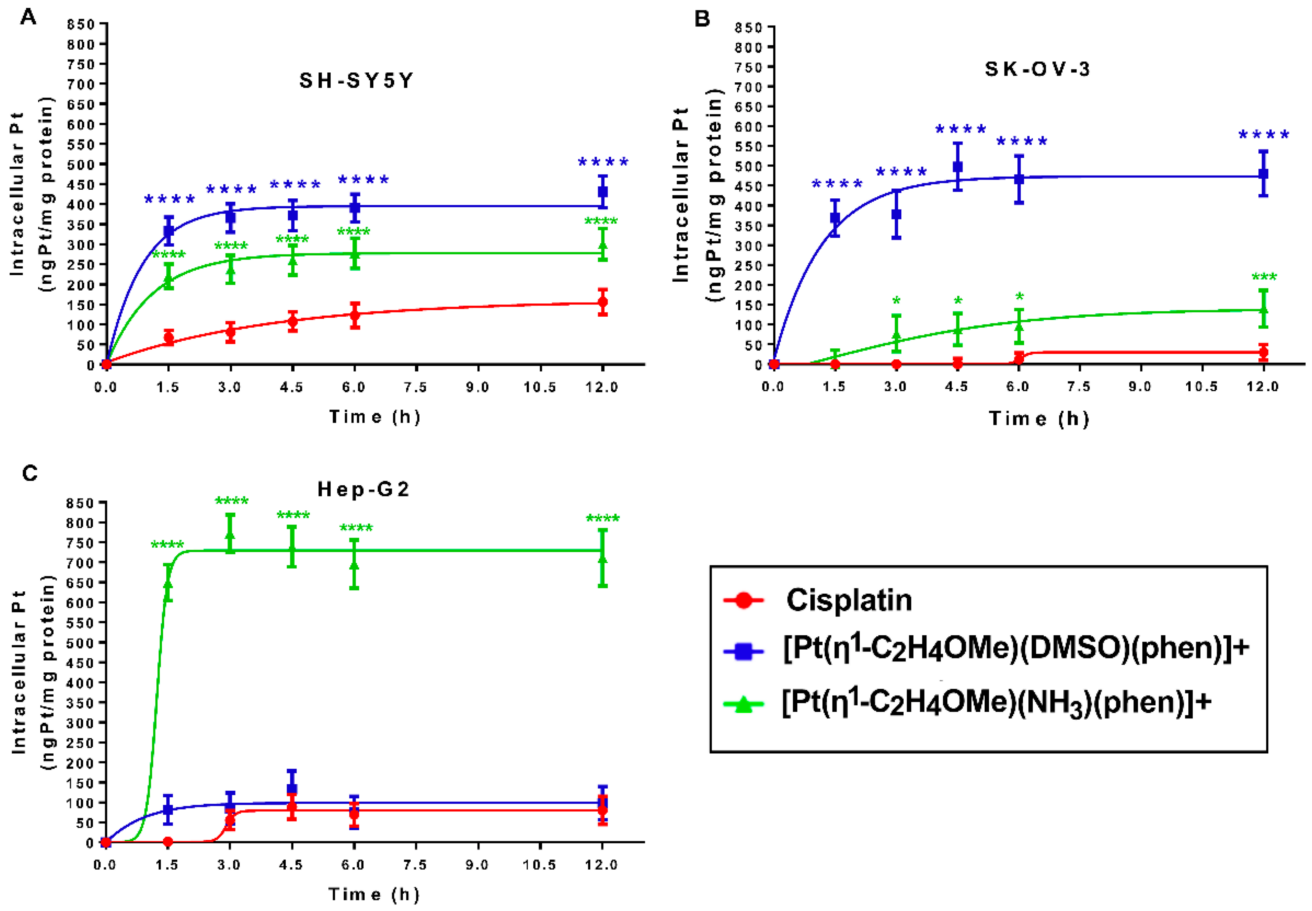
Intracellular uptake after [Pt(η^1^-C_2_H_4_OMe)(DMSO)(phen)]^+^, [Pt(η^1^-C_2_H_4_OMe)(NH_3_)(phen)]^+^, and cisplatin treatments in SH-SY5Y (**A**), SK-OV-3 (**B**) and Hep-G2 (**C**) cell lines. The total intracellular accumulation was determined by ICP-AES; cells were continuously exposed to 100 μM of each Pt compound for 1.5–12 h. Each point represents the means ± S.D. of three different experiments and is indicated as ng of Pt(II)/mg of protein. Asterisks indicate values that are significantly different (* *p* < 0.05; *** *p* < 0.001; **** *p* < 0.0001) from those of cisplatin at the same concentration and time point.

Total intracellular Pt(II) content was evaluated in Hep-G2, SH-SY5Y and SK-OV-3 after treatment with 100 μM of cisplatin, [Pt(η^1^-C_2_H_4_OMe)(NH_3_)(phen)],^+^ (**2**) and [Pt(η^1^-C_2_H_4_OMe)(DMSO)(phen)]^+^ (**3**) or by ICP-AES after 1.5, 3, 4.5, 6, and 12 h of incubation. In accordance with what has been found for cisplatin [50] and for [Pt(*O*,*O*′-acac)(γ-acac)(DMS)] [42,51,52], the here considered new Pt(II) compounds seemed to enter cells via a passive or facilitated passive transport very quickly, and the cellular accumulation of **3** was higher than that of both cisplatin and **2** (Figure 7 and Table S2). Thus, the high cytotoxicity of the [Pt(η^1^-C_2_H_4_OMe)(DMSO)(phen)]^+^ complex may be related to a high intracellular uptake with respect to cisplatin, both in SH-SY5Y (about 4.5-fold) (Figure 7A) and SK-OV-3 cells (about 18-fold) (Figure 7B). However, no correlation between the cytotoxicity and the intracellular uptake of the [Pt(η^1^-C_2_H_4_OMe)(NH_3_)(phen)]^+^ complex was found; in fact, this compound did not decrease cell viability, although its intracellular uptake was greater than that of cisplatin in both SH-SY5Y (about four-fold) (Figure 7C) and SK-OV-3 cells (about three-fold) (Figure 7B). In addition, although complex **2** accumulated considerably, it showed no significant cytotoxic effects in Hep-G2 cells (Figure 6). Therefore, these Pt(II)-based compounds exhibited very different uptake profiles and cytotoxicity levels in the three considered cell lines, suggesting diversified biological effects, thus making them potentially tissue- and tumor-specific drugs.

## 4. Conclusions

Several strategies have been developed to solve some of the problems related to the clinical application of cisplatin, since its approval. Greater spectrum of efficacy, lower side effects, and enhanced water solubility have been constantly targeted by the researchers in the field [1,2,3,4,16]. In this work, specific platinum drugs in the form of cationic solvento species and, therefore, possible substrates for organic cation transporters (OCT) were studied. In particular, two novel water-soluble organometallic cationic species, [Pt(η^1^-C_2_H_4_OMe)(NH_3_)(phen)]^+^ (**2**) and [Pt(η^1^-C_2_H_4_OMe)(DMSO)(phen)]^+^ (**3**), were obtained from [PtCl(η^1^-C_2_H_4_OMe)(phen)] by clorido substitution, using the Zeise’s anion, [PtCl_3_(η^2^-C_2_H_4_)]^−^ as starting material. The new cationic complexes (**2**, **3**) were spectroscopically characterized, and their cellular uptake and cytotoxicity evaluated, in comparison with cisplatin, for a range of tumor cell lines. The Pt(II)-based drugs exhibited very different uptake profiles and cytotoxicity levels in the considered cell lines, suggesting diversified biological effects. The DMSO complex (**3**) showed general higher cytotoxicity, with respect to the amino species **2**, exhibiting also lower IC_50_ values with respect to cisplatin in almost all cell lines starting from the first hours of treatment, particularly in neuroblastoma and adenocarcinoma cell lines. It is also worth pointing out that cellular uptake did not appear to be directly related to cytotoxicity as (**a**) there was a significant uptake of the DMSO complex **3** in hepatocarcinoma cells without this compound being more effective than cisplatin; (**b**) the uptake of the NH_3_ complex was elevated in liver cells without demonstrating important cytotoxic effects; (**c**) the uptake of the new complexes was always greater than that of cisplatin and strain-dependent in the investigated cells, even if their cytotoxicity was not always greater than that of cisplatin. The different behavior of the two [Pt(η^1^-C_2_H_4_OMe)(L)(phen)]^+^ complexes could be related to the much higher lability of the DMSO derivative with respect to that with NH_3_, with consequent easier formation of active solvento species containing the Pt(phen) moiety. This aspect differentiates complex **3** from cisplatin where the presence of DMSO can inhibit the formation of the antitumor active aquo species, with a consequent general reduction of the observed cytotoxicity [46,49,53]. More in-depth studies will be conducted in order to determine the mechanism of action of the [Pt(η^1^-C_2_H_4_OMe)(DMSO)(phen)]^+^ (**3**) complex and its metabolic alterations in the cell lines more sensitive to its antiproliferative activity. Nevertheless, the new Pt(II)-based compounds exhibited specific uptake profiles and different cytotoxicity levels in the considered cell lines, suggesting the occurrence of diversified biological effects. Therefore, a potential use of the obtained species as models for tissue- and tumor-specific drugs design is also envisaged.

## Figures and Tables

**Table 1:** Cell cultures.

Cell Lines	Disease
Caco-2	Colorectal adenocarcinoma
HeLa	Endocervical adenocarcinoma
Hep-G2	Hepatocellular carcinoma
MCF-7	Breast adenocarcinoma
MG-63	Osteosarcoma
SH-SY5Y	Neuroblastoma
SK-OV-3	Ovarian adenocarcinoma
ZL-55	Pleural epithelioid mesothelioma

## Supplementary Materials

The following are available online at https://www.mdpi.com/article/10.3390/pharmaceutics13050642/s1, Figure S1. Water hydrolysis of the [Pt(η^1^-C_2_H_4_OMe)(DMSO)(phen)]^+^ complex **3** followed by ^1^H NMR spectroscopy. Figure S2. ^195^Pt NMR spectrum of the [{Pt(μ-OH)(phen)}_2_]^2+^ solvento species. Figure S3. Cell viability assay after 0.725% DMSO treatments in SK-OV-3, Hep-G2, and SH-SY5Y cell lines. Table S1. The IC_50_ after [Pt(η^1^-C_2_H_4_OMe)(DMSO)(phen)]^+^ and cisplatin treatment in eight human cancer cell lines. Table S2. Pt total intracellular accumulation, determined by ICP-AES, in SH-SY5Y, SK-OV-3, and Hep-G2 cell lines, exposed to 100 μM of each of the **2** and **3** Pt compounds for 1.5–12 h.
