# Supplementary material for: Synthesis and Evaluation of the Cytotoxic Activity of Water-Soluble Cationic Organometallic Complexes of the Type [Pt(η1-C2H4OMe)(L)(Phen)]+ (L = NH3, DMSO; Phen = 1,10-Phenanthroline)
Source: Pharmaceutics. 2021 Apr 30;13(5):642. doi: 10.3390/pharmaceutics13050642 (PMC8147149; doi:10.3390/pharmaceutics13050642)
Supplement: Supplementary file 1 [file pharmaceutics-13-00642-s001.zip › pharmaceutics-1183804-supplementary.pdf]

pharmaceutics

MDPI

Supplementary Materials: Synthesis and Evaluation of the Cytotoxic Activity of Water-Soluble Cationic Organometallic Complexes of the Type [Pt(η1-C_2H_4OMe)(L)(Phen)]+ (L = NH_3, DMSO; Phen = 1,10-Phenanthroline)

Federica De Castro, Erika Stefàno, Danilo Migoni, Giorgia N. Iaconisi, Antonella Muscella, Santo Marsigliante, Michele Benedetti and Francesco P. Fanizzi

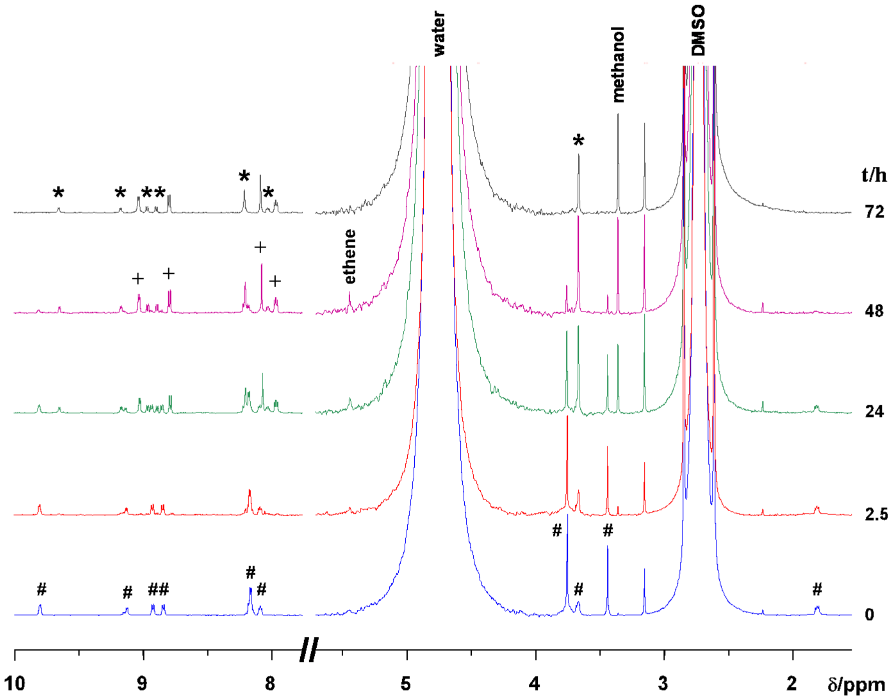

Figure S1. Hydrolysis of the [Pt(η^1-C_2H_4OMe)(DMSO)(phen)]Cl (**3**, #) complex, dissolved in a neutral water/DMSO mixture (D_2O/DMSO = 90/10), followed by ^{1}H NMR spectroscopy. In the 0-72 h time interval, it is shown a progressive formation of the $[Pt(DMSO)(OH)(phen)]^+$ (*) and $[\{Pt(\mu\text{-}OH)(phen)\}_2]^{2+}$ (+) species, as a consequence of complex **3** hydrolysis.

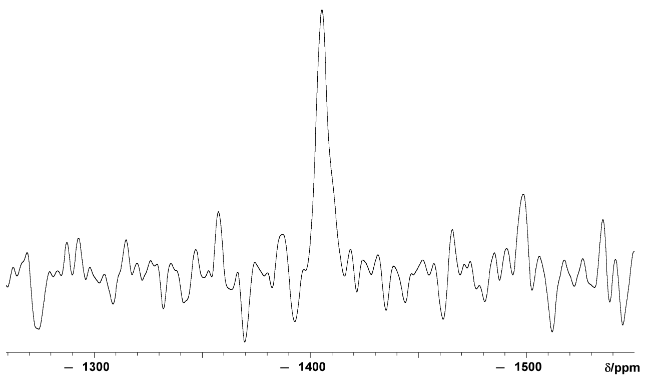

Figure S2. ^{195}Pt NMR signal revealed for a D_2O solution of the [Pt(η^1-C_2H_4OMe)(DMSO)(phen)]Cl ·2DMSO (**3**·2DMSO) complex, left to hydrolyze in D_2O for three days at room temperature. The observed ^{195}Pt NMR signal is attributable to formation of the $[\{Pt(\mu\text{-}OH)(phen)\}_2]^{2+}$ dimeric hydrolysis product [1].

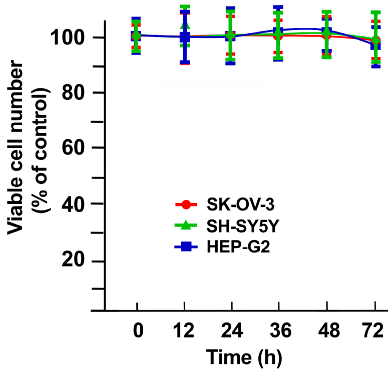

Figure S3. Viability of cell lines was evaluated in the incubation medium and 0.725% DMSO for 12, 24, 48, and 72 h. The data are means ± S.D. of three different experiments run in eight replicates and are presented as percent of control.

Table S1. IC_{50} values measured after $[Pt(\eta^1\text{-}C_2H_4OMe)(DMSO)(phen)]^+$ and *cisplatin* treatment, at different concentrations (0.1, 1, 10, 100 and 200 μM) and time intervals, for the eight tested human cancer cell lines.

	IC50 (μM), 12 h		IC50(μM), 24 h		IC50(μM), 48 h		IC50(μM), 72 h	
**Cell lines**	***Cisplatin***	**$[Pt(\eta^1\text{-}C_2H_4OMe)(DMSO)(phen)]^+$**	***Cisplatin***	**$[Pt(\eta^1\text{-}C_2H_4OMe)(DMSO)(phen)]^+$**	***Cisplatin***	**$[Pt(\eta^1\text{-}C_2H_4OMe)(DMSO)(phen)]^+$**	***Cisplatin***	**$[Pt(\eta^1\text{-}C_2H_4OMe)(DMSO)(phen)]^+$**
**Caco-2**	>200	>200	>200	>200	176.8 ± 9.2	115.0 ± 9.5	81.0 ± 5.4	67.0 ± 6.4
**HeLa**	>200	69.8 ± 5.0	81.5 ± 7.9	74.7 ± 5.9	6.3 ± 1.2	62.9 ± 5.4	2.1 ± 0.7	17.9 ± 2.8
**Hep-G2**	>200	>200	>200	178.5 ± 9.3	137.0 ± 7.8	160.7 ± 7.4	79.8 ± 6.9	108.9 ± 6.5
**MCF-7**	>200	76.4 ± 6.1	>200	61.7 ± 5.3	115.0 ± 9.8	51.3 ± 4.2	56.3 ± 4.6	39.5 ± 3.5
**Mg-63**	>200	62.0 ± 5.1	65.6 ± 5.2	57.6 ± 4.2	35.8 ± 3.9	46.1 ± 4.2	21.4 ± 4.9	41.1 ± 3.1
**SH-SY5Y**	>200	56.9 ± 7.7	50.0 ± 9.6	45.8 ± 7.8	20.8 ± 4.8	46.1 ± 4.2	5.4 ± 2.6	15.1 ± 2.6
**SK-OV-3**	>200	92.1 ± 7.8	130 ± 8.9	62.8 ± 6.6	69.8 ± 6.9	48.2 ± 5.9	41.7 ± 4.6	37.5 ± 3.2
**ZL-55**	>200	139.1 ± 6.8	53.3 ± 5.0	79.2 ± 5.4	36.7 ± 3.1	41.1 ± 3.4	3.8 ± 1.2	40.1 ± 3.5

Table S2. Total Pt intracellular accumulation, determined by ICP-AES, in SH-SY5Y, SK-OV-3 and Hep-G2 cell lines, exposed to 100 μM of each Pt-compound for the indicated time. Each point represents the means ± S.D. of three different experiments and are indicated as ng of Pt(II)/mg of protein.

Cell Lines	1.5 h	3.0 h	4.5 h	6.0 h	12 h
		Cisplatin **(ngPt/mg protein)**			
Hep-G2	2.0 ± 10.5	55.5 ± 23.8	88.8 ± 31.0	69.1 ± 28.3	80.0 ± 35.0
SH-SY5Y	67.1 ± 18.0	80.5 ± 24.2	107.3 ± 23.4	122.3 ± 29.8	155.9 ± 31.4
SK-OV-3	0	0	0	12.1 ± 16.70	30.0 ± 20.2
		**$[Pt(\eta^1\text{-}C_2H_4OMe)(DMSO)(phen)]^+$ (ngPt/mg protein)**			
Hep-G2	81.3 ± 36.3	84.5 ± 39.1	132.7 ± 46.1	75.2 ± 39.7	98.0 ± 41.5
SH-SY5Y	332.8 ± 34.6	365.4 ± 35.5	371.1 ± 37.9	389.9 ± 34.7	430.5 ± 40.4
SK-OV-3	368.4 ± 45.2	377.7 ± 58.9	497.6 ± 58.1	465.8 ± 58.8	480.0 ± 55.6
		**$[Pt(\eta^1\text{-}C_2H_4OMe)(NH_3)(phen)]^+$ (ngPt/mg protein)**			
Hep-G2	649.3 ± 44.8	771.8 ± 46.7	738.7 ± 49.6	695.4 ± 59.6	711.0 ± 69.8
SH-SY5Y	220.6 ± 30.1	237.2 ± 35.0	260.0 ± 37.1	277.4 ± 37.5	300.1 ± 30.3
SK-OV-3	0	77.0 ± 45.0	87.6 ± 40.2	95.8 ± 42.1	140.0 ± 45.8

Reference

1. Wimmer, S.; Castan, P.; Wimmer, F.L.; Johnson, N.P. Preparation and Interconversion of Dimeric Di-μ-Hydroxo and Tri-μ-Hydroxo Complexes of Platinum(II) and Palladium(II) with 2,2′-Bipyridine and 1,10-Phenanthroline. *J. Chem. Soc. Dalton Trans.* **1989**, *3*, 403–412, doi:10.1039/DT9890000403.
